# Thoracic Pedicle Morphometry of Dry Vertebral Columns in Relation to Trans-Pedicular Fixation: A Cross-Sectional Study From Central India

**DOI:** 10.7759/cureus.8148

**Published:** 2020-05-16

**Authors:** Virendra Verma, John A Santoshi, Vaibhav Jain, Manmohan Patel, Manish Dwivedi, Manoj Nagar, Rajkumar Selvanayagam, Dharm Pal

**Affiliations:** 1 Orthopaedics, All India Institute of Medical Sciences, Bhopal, IND; 2 Anatomy, All India Institute of Medical Sciences, Bhopal, IND

**Keywords:** pedicle morphometry, spine, thoracic, instrumentation, pedicle screw, india

## Abstract

Introduction

Trans-pedicular screw fixation is one of the main modalities of spinal instrumentation today. It is particularly challenging in the thoracic spine due to the narrow pedicle dimensions especially in the upper and mid-thoracic levels. We aimed to study the anatomical variations like pedicle dimensions and angulation in transverse and sagittal planes.

Material and methods

We conducted an anatomical investigation on 20 dry vertebral columns (14 male and six female), from T1 to T12 levels. The measurements included pedicle width, height, and transverse and sagittal angles of the pedicle. Numerical variables were summarized using mean and standard deviation.

Results

T12 vertebra was found to have the widest pedicle width (mean 7.89 ± 0.70 mm) and the widest pedicle height (mean 15.45±0.78 mm) while T5 vertebra (mean 3.65±0.40 mm) had the narrowest pedicle width. T1 vertebra had the maximum transverse angle of the pedicle (mean 30.37±2.56 degree); whereas, T2 vertebra had the maximum sagittal angle (mean 19.22±2.24 degree).

Conclusion

We have reported detailed pedicle measurements including their angulation for the thoracic spine in dry vertebral columns of central India. The pedicles are directed more medially from T1 to T10 levels and are almost neutral at T11 and T12 levels. These findings would not only be of immense help to the spinal surgeons but also help in designing implants and instrumentations specific for the thoracic spine for the central Indian population as well as aiding surgeons to perform more precise and, therefore, safe surgical procedures.

## Introduction

Pedicles of the vertebrae are the short, thick, cylindrical bony processes that project posteriorly from the superior part of the vertebral body and fuse with the laminae to form the neural arch. These are situated between the transverse process and the spinous process. In the thoracic spine, the spinal cord and segmental nerves constitute the content of the spinal canal. The contents of the spinal canal are at risk of injury during spinal instrumentation.

Earlier, laminar hooks and sublaminar wires were used for spinal instrumentation. These had lesser strength and carried the risk of encroachment into the spinal canal as they held the lamina for fixation to the spine. Posterior spinal instrumentation through pedicles has been utilized for the past six decades [1]. Theoretically, with the use of pedicle screws, which are placed into the vertebral body through the pedicles, there is no invasion into the spinal canal and the fixation strength is greater [2]. The posterior spinal instrumentation through pedicles is frequently used for the management of vertebral fractures, degenerative spine, spondylolisthesis and correction of deformity [1]. They can also be used post-laminectomy where laminar hooks and sublaminar wires cannot be used. Pedicle screw malpositioning has resulted in complications like pedicle penetration, fracture of pedicle, neurological irritation and cerebrospinal fluid leakage. To prevent these complications, precise screw positioning is of prime importance. The surgeons contemplating pedicle screw fixation need to have a thorough knowledge of the spinal anatomy and should be able to identify and localize bony as well as neural structures precisely. This requires a combination of directly visualized bony anatomy, proprioceptive feedback, preoperative planning, and intraoperative radiological imaging [1-5].

Racial variations in pedicle morphology in different ethnic groups and populations are well documented as are the differences between males and females [6-8]. The standard pedicle screws are available from 4.5 mm to 8 mm diameter and are based on the morphometric analysis of the Caucasian population. Since variation in pedicle morphology could affect the quality of fixation and possible risk of complications, it would be of great interest to determine if the available screws are suitable for the central Indian population. We aimed to study the anatomical variations like pedicle dimensions and angulation in transverse and sagittal plane to facilitate safe pedicle screw insertion in the thoracic spine while preventing neurological complications.

## Materials and methods

**Figure 1:**
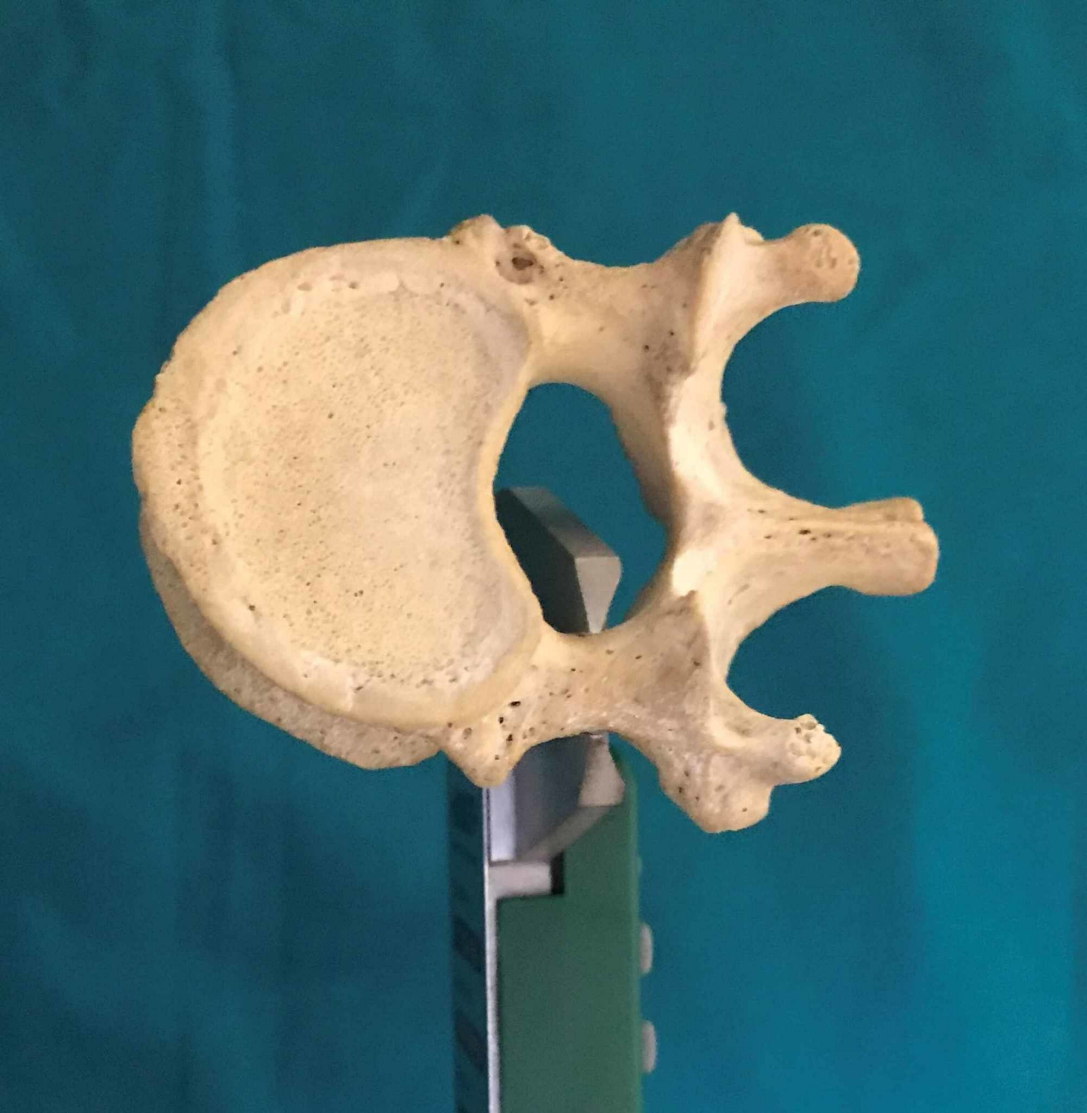
Pedicle width

**Figure 2:**
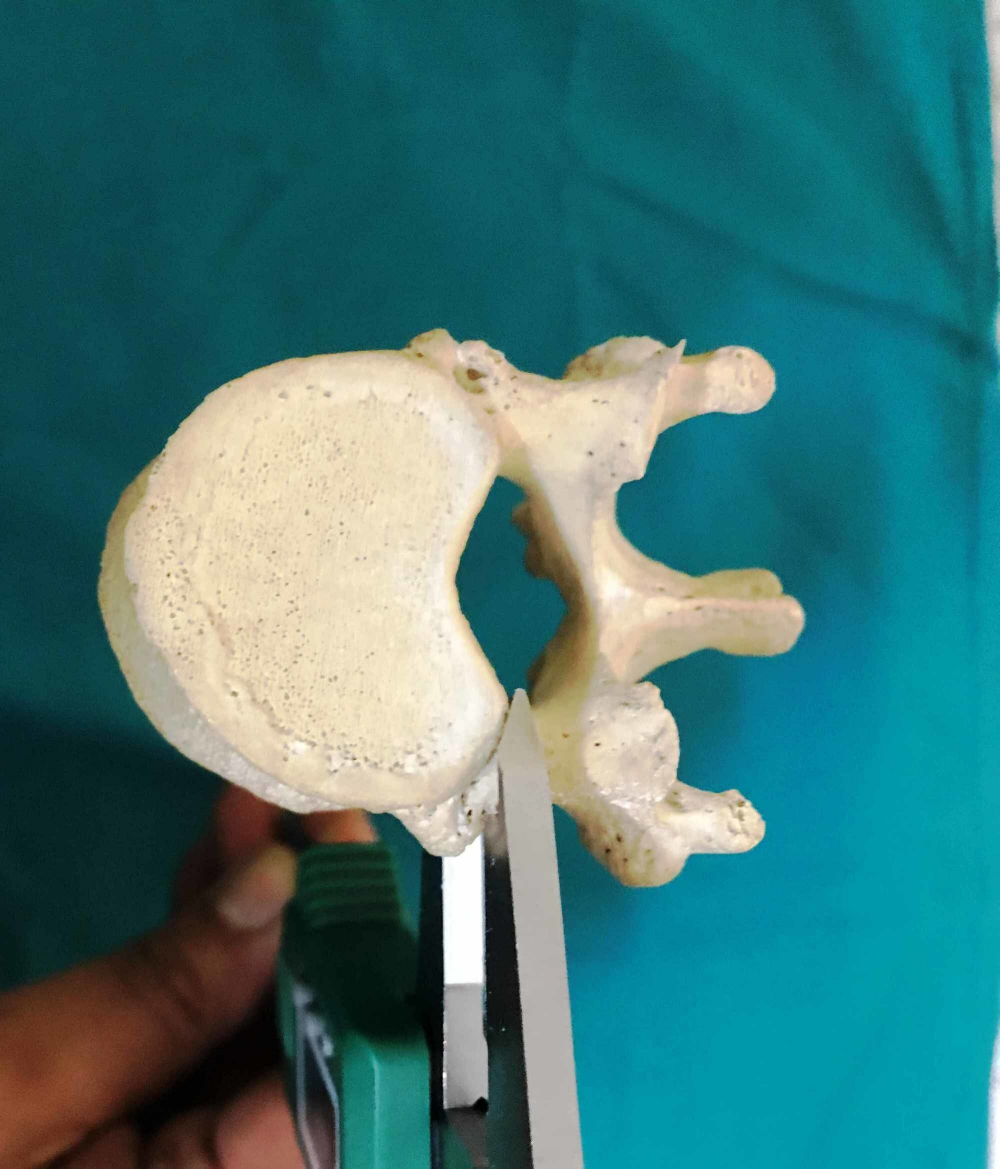
Pedicle height

**Figure 3:**
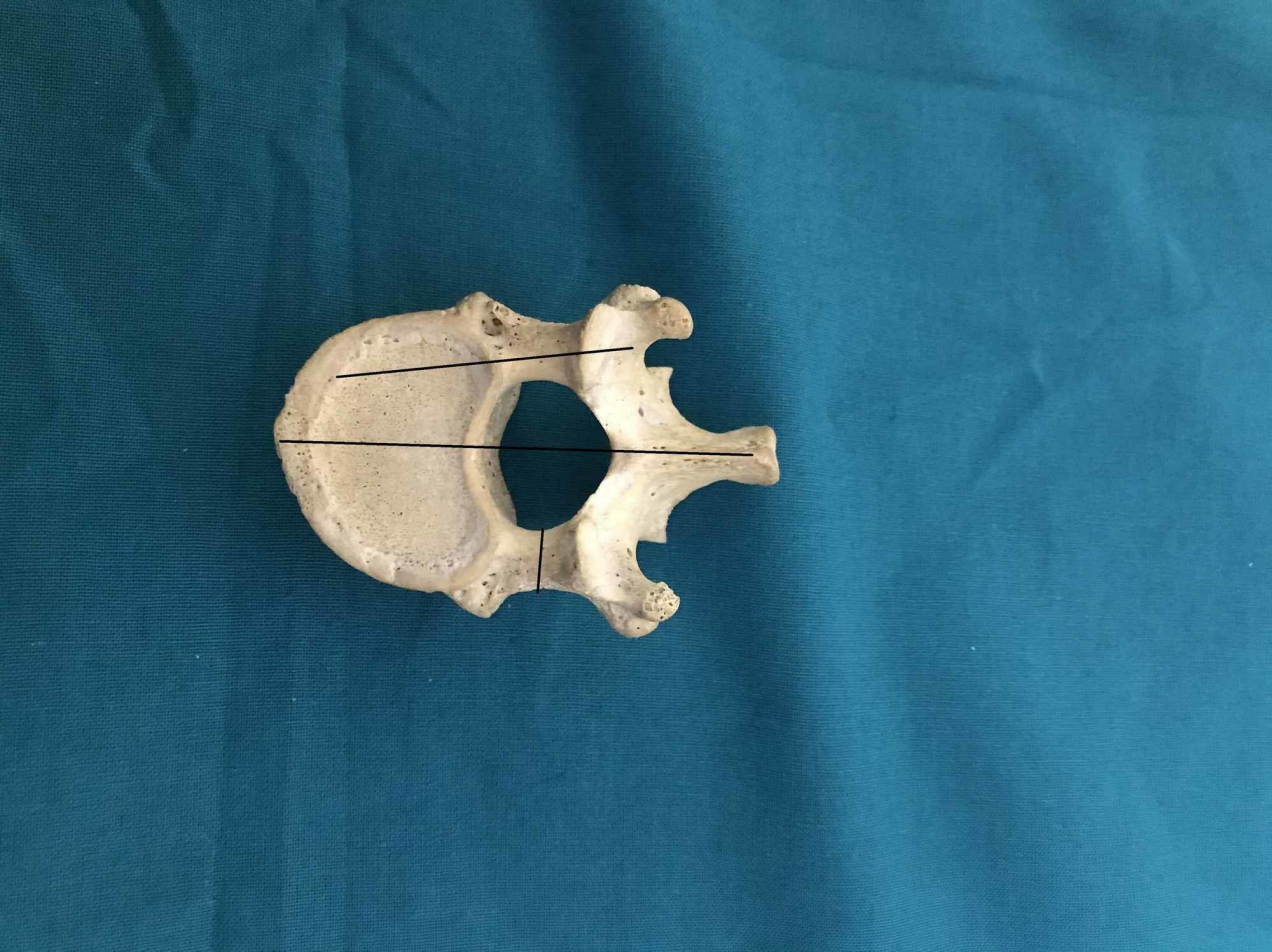
Transverse pedicle angle and pedicle width

**Figure 4:**
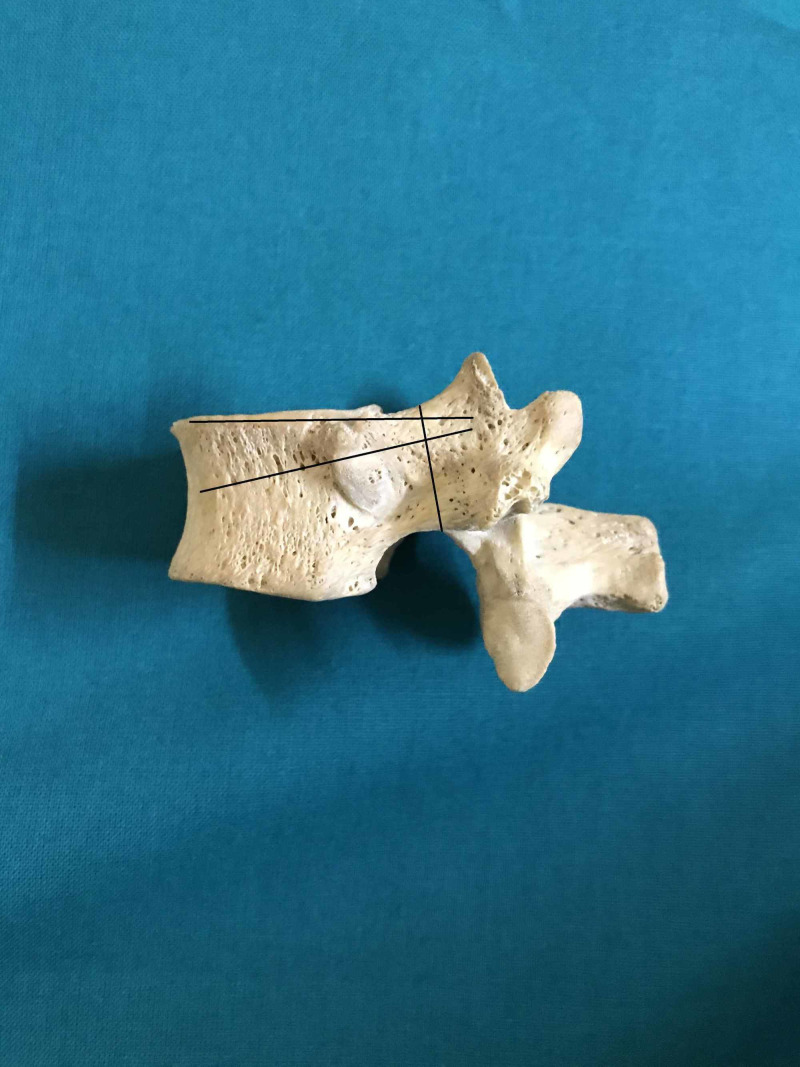
Sagittal angle and pedicle height

We performed an anatomic investigation on the dry vertebral column after institutional research and review board approval. Twenty adult dry vertebral columns (14 male and six female) were available for the study. All the vertebral columns were free of prior trauma or visible deformity. The morphometric measurements with respect to dimensions of pedicles of thoracic vertebrae were done. The measurements included pedicle width (transverse external diameter), pedicle height (sagittal external diameter), transverse and sagittal angles of the pedicle [9]. The measurements were performed by using Vernier calipers and standard goniometer. The narrowest transverse external diameter of the pedicle was recorded at the pedicle width (Figure 1). The narrowest sagittal external diameter of the pedicle was recorded as the pedicle height (Figure 2). The transverse angle of pedicles was defined as the angle between the pedicle axis and a line parallel to the vertebral midline measured in the transverse plane (Figure 3). The sagittal angle of the pedicle was defined as the angle between the pedicle axis and the superior border of the vertebral body in the sagittal plane (Figure 4).

Statistical analysis

The measurements were tabulated, and the data were analyzed using the Excel 2010 program (Microsoft®, Redmond, Washington, USA). Numerical variables were summarized using mean and standard deviation.

## Results

Both pedicle width and pedicle height varied between individual vertebral columns and between levels, although these dimensions did not differ significantly between the right and left sides.

Pedicle width (transverse external diameter) - decreased progressively from T1 to T5 levels and then increased gradually to T12 level. Among all the thoracic vertebrae, T12 was found to have the widest pedicle width (mean 7.89 ± 0.70 mm); whereas, the narrowest pedicle width was at T5 (mean 3.65 ± 0.40 mm) (Table 1).

Pedicle height (sagittal external diameter) - was narrowest at T1 level (mean 8.77 ± 0.69 mm) and it progressively increased up to the T12 level (mean 15.45 ± 0.78 mm) and it was maximum at that level for the thoracic spine (Table 2).

The transverse angle of pedicle - a constant decrease in transverse angle of pedicles was observed from T1 to T12 level. The highest angle was seen at T1 (mean 30.37 ± 2.56 degree) and least at T12 (mean 7.19 ± 0.87 degree) (Table 3).

The sagittal angle of pedicle - T2 was observed to have the highest sagittal angle (mean 19.22 ± 2.24 degree) while the least sagittal angle was observed at T12 (mean 9.55 ± 0.88 degree) (Table 4).

**Table 1:** Comparison of transverse diameter (pedicle width in mm) with other studies

	Datir & Mitra [10]	Hou et al. [11]	Scoles et al. [12]	Chaynes et al. [13]	Vaccaro et al. [14]	Kim et al. [15]	Singh et al. [16]	Kaur et al. [17]	Current study
	(NN =18)	(N = 25)	(N = 25)	(N =14)	(N = 24)	(N = 42)	(N=100)	(N= 50)	(N=20)
T1	7.3		7.3	8.3		8.1	7.72	9.27	7.69
T2	6.3			6.5		6.1	6.22	7.5	5.7
T3	5.2		3.9	5.9		4.6	5.03	6	4.19
T4	4.8			5.4	4.5	4.2	4.53	4.5	3.69
T5	4.7			4.9	4.4	4.3	4.22	5	3.65
T6	5		3.5	5.1	4.6	4.7	4.58	5.5	3.88
T7	5.4			5.7	4.7	4.8	4.82	6	4.47
T8	5.4			6.4	5.1	5.1	4.82	6.32	4.79
T9	5.9	6	3.9	6.4	5.8	5.2	5.33	6.28	5.35
T10	6.7	7		7.4	6.7	6.3	6.1	6.54	5.8
T11	8.2	8.6		9.3	8	7.9	7.36	7.84	7.39
T12	8.7	8.8	7.4	8.9	7.8	7.9	7.94	8.31	7.89

**Table 2:** Comparison of sagittal diameter (pedicle height in mm) with other studies

Serial No.	Datir & Mitra [10]	Hou et al. [11]	Vaccaro et al. [14]	Scoles et al. [12]	Singh et al. [16]	Current Study
	(N = 18)	(N = 25)	(N = 8–24)	(N = 25)	( N= 100)	(N=20)
T1	9.4			9.2	8.6	8.77
T2	12.1				10.58	10.32
T3	12.2			11.8	11.39	10.47
T4	11.8		10.1		11.09	10.43
T5	11.6		10.6		10.86	10.38
T6	11.7		10.1	11.5	10.85	10.13
T7	12.5		10.8		11.2	10.46
T8	13.2		11.1		11.75	10.94
T9	14.4	12.5	12.3	12.9	12.81	12.41
T10	16.6	14.4	14.1		14.22	13.73
T11	17.7	16.4	15		15.55	14.94
T12	18.7	17.1	14.7	16	15.53	15.45

**Table 3:** Comparison of transverse pedicle angle (in degrees) with other studies

S.No.	Datir & Mitra [10]	Scoles et al. [12]	Chaynes et al. [13]	Vaccaro et al. [14]	Zindrick et al. [18]	Singh et al. [16]	Kaur et al. [17]	Current Study
	(N = 18)	(N =25)	(N = 14)	(N = 24)	(N = 42)	(N=100)	(N=50)	(N= 20)
T1	30	29.8	27.5		27	31.8	35.4	30.37
T2	19		17.3		20	25.8	26.21	26.27
T3	12	15.3	13		15	20.79	20.01	24.43
T4	6		8.1	14	13	8.12	19.06	17.4
T5	4		6.8	13	9	15.5	16	23.32
T6	3	10.2	6.7	9	10	13.06	14.38	15.84
T7	1		7.2	7	9	12.25	11.82	12.71
T8	1		7.1	7	8	11.22	12.29	14.35
T9	1	9.2	0.9	7	8	10.09	11.21	10.96
T10	1		7.7	4	5	8.78	8.7	8.42
T11	0		0.8	1	1	-1.4	-2.3	8.07
T12	0	9.5	2	0	4	-10.01	-9.8	7.19

**Table 4:** Comparison of sagittal angle (in degrees) with other studies

S. no.	Datir & Mitra [10] (N = 18)	Zindrick et al. [18] (N = 42)	Singh et al. [16] (N=100)	Current study (N=20)
T1	9.6	12.6	15.03	14
T2	11.8	17.5	16.9	19.2
T3	10.4	17.3	17.6	16.5
T4	8.9	16.3	16.7	17.5
T5	9.4	15	16.2	15.4
T6	8.2	15	15.2	16.8
T7	9.2	15.7	16.2	14.4
T8	8.6	16.6	15.6	13.8
T9	7.6	16	14.8	12.7
T10	5.5	16.8	10.7	13.1
T11	6.3	15.4	7.77	12.4
T12	8.5	11.6	3.92	9.5

## Discussion

The pedicles are the strongest part of a vertebra and are made up entirely of cortical bone with a small core of cancellous bone. They act as a strut to transmit forces between the vertebral body and the neural arch [14]. According to Pal and Routal who studied the role of neural arch in weight transmission using morphometric methods, the thoracolumbar spine consists of two vertical running columns which are involved in load transmission - the anterior column is formed by the vertebral body and intervertebral disc while the posterior column is formed by successive articulations of neural arch elements (facet joints, laminae, and posterior ligamentous complex) [19]. The relative magnitude of compressive force passing through the body and neural arch alters with the change of curvature at a cervicothoracic and thoracolumbar junction along the vertebral column. The transfer of compressive forces between the body and neural arch takes place through the pedicle, which acts as a beam connecting the two columns. The pedicles transmit both tensile and bending forces which include gravitational loads as well as muscular movements.

While transpedicular spinal fixation was first described by Boucher in the 1950s, it is the work of Louis, Roy-Camille, and Saillant in the past four decades that trans-pedicular fixation has become a popular method of spinal instrumentation [1,20]. To prevent injury to the neural structures, a safe pathway through the pedicle is important. This requires thorough knowledge of the anatomy of bony as well as neural structures [1,2]. The rigidity and rotational stability of pedicular screw fixation systems are determined by the pull-out strength and depth of insertion into the vertebral body; the screw diameter should fill more than 70% of the pedicle diameter, the wider the screw, the stronger is the fixation [21]. Many authors have described the morphometric aspects of the thoracic spine and the details of the pedicle sizes and dimensions by means of computed tomography (CT) scan, plain radiographs, direct specimen measurement and quantitative 3-dimensional anatomic techniques [6,8-10,12,13,17,19,20].

We measured the dimensions in twenty dry vertebral columns from T1 to T12 vertebrae. The levels at risk for pedicular breach were observed to be at T3 to T8 levels. The pedicle width gradually decreased from T1 to T5 and then, started increasing from T6 toT12 in the present study. Similar trend was also reported in other studies [6,8,10,13,16,17]. This pattern of change in the size of the pedicles may be due to transition from a more mobile cervicothoracic junction to a relatively fixed mid-thoracic region, and again, to a mobile thoracolumbar junction putting differential stress on the facet joints and pedicles. Since these pedicles have smaller diameters it is suggested to plan pedicle screw trajectory and size preoperatively using CT scan. Knowledge of pedicle morphometry would add to the comfort level of the spinal surgeon, especially, one who is in the early stages of his or her career.

Specific differences in the pedicle dimensions between males and females have been reported in the literature. Kim et al. reported differences in pedicle width between males and females at T10, L3, and L5 levels while Hou et al. reported differences only at T12 level, wherein the male pedicles were found to be larger [11,15]. We did not study the sex-differences.

Our findings are in concurrence with other authors in that the risk of a cortical breach during pedicle screw insertion is highest from T3 to T8 levels using conventional techniques [17,21-23]. Various modifications like the ‘in-out-in’ technique, medial margin targeting method, pedicle or transverse process hooks, extra-pedicular screw, cannulated screw, laminar screw, and the cortical bone trajectory method have been studied as safe alternatives for thoracic spine fixation [20,24-29]. Use of custom or patient-specific implants and instrumentation using additive manufacturing technology is also recommended for these levels along with computer and spinal navigation assisted spinal instrumentation, however, these have the disadvantage of increased the cost of treatment and non-availability [5,30].

In the present study, transverse pedicle angles from T1 to T10 vertebrae were found to be more compared to studies performed on Caucasians. Our findings of the pedicles being angulated medially, postero-anteriorly, decreasing in the cephalo-caudad direction from T1 to T4, increasing slightly at T5, and then decreasing trend from T5 to T12, are similar to other studies from India [10,16,17]. The Caucasian studies show a gradually decreasing trend from T1 to T12 [19]. The pedicle angulation in the transverse plane was closer to the neutral plane at T11 and T12 levels in some studies while it was 8.07o and 7.19o cephalad in the present study [10,13,16,17] (Table 3).

In the sagittal plane, we found the pedicles were angulated in the cephalad direction and the angulation gradually decreased from T2 caudad; 19.22o at T2 to 9.55o at T12. These findings are comparable to studies by Datir and Mitra and Zindrick et al. who reported maximum sagittal angulation at T2 level (11.8o and 17.5o respectively) while Singh et al. reported the maximum sagittal angulation at T3 level (17.6o) [10,16,18]

The general trend of morphological measurement of the pedicle dimensions in the present study was comparable to the findings of other authors from India, and also, to the Chinese population; these measurements were lower than those of the Caucasians [6,10,11,20]. These differences could be attributed to the larger physique of the Caucasians.

Limitations of the study

We recognise a few limitations of the study. Since we studied dry vertebral columns, we could not study the sex and age-related differences. Secondly, performing actual pedicle screw insertion in cadavers and looking for pedicle breach would have provided additional information. However, we could not perform this due to the lack of available intact cadaveric vertebral columns as these are used for undergraduate teaching at our institute.

## Conclusions

Pedicle instrumentation in thoracic vertebrae provides little margin of safety due to its morphometric features. The anatomical variation in different population groups should be considered while using thoracic pedicle screws. The findings of our study would be of immense help to the spinal surgeons especially those in the early phase of their career in planning and placement of thoracic pedicle screws. The results of the study could help in designing implants and instrumentations specific for the thoracic spine for central Indian population aiding surgeons to perform more precise and, therefore, safe surgical procedures.
